# The impact of transcutaneous tibial nerve stimulation on brain function in patients with overactive bladder: a prospective longitudinal self-controlled study

**DOI:** 10.3389/fnhum.2026.1849595

**Published:** 2026-08-28

**Authors:** Ziyu Wang, Yuansong Xiao, Xiangjun Lyu, Han Deng, Chen Li, Dongsheng Shang, Linquan Jin, Limin Liao, Yun Guo, Xing Li

**Affiliations:** 1 Department of Urology, Rehabilitation School of Capital Medical University, China Rehabilitation Research Center, Beijing, China; 2 Department of Urology, General Hospital of Southern Theater Command, Guangzhou, China; 3 Department of Urology, The Third Medical Center of Chinese PLA General Hospital, Beijing, China; 4 Department of Rehabilitation, The Second Affiliated Hospital and Yuying Children’s Hospital of Wenzhou Medical University, Wenzhou, China; 5 Department of Rehabilitation Medicine, Beijing Tsinghua Changgung Hospital, School of Clinical Medicine, Tsinghua Medicine, Tsinghua University, Beijing, China

**Keywords:** fractional amplitude of low-frequency fluctuations, functional connectivity, functional magnetic resonance imaging, overactive bladder, transcutaneous tibial nerve stimulation

## Abstract

**Objective:**

This study aimed to explore brain activity and functional connectivity changes associated with transcutaneous tibial nerve stimulation (TTNS) in patients with neurogenic overactive bladder (OAB), alongside changes in clinical symptoms.

**Methods:**

This prospective longitudinal self-controlled trial, conducted at the China Rehabilitation Research Center from 2024 to 2025, enrolled 15 adults with symptom-defined neurogenic OAB associated with spinal cord injury or multiple sclerosis. Resting-state functional magnetic resonance imaging (fMRI) was performed before and after standardized TTNS under full- and empty-bladder conditions. The primary imaging outcomes were fractional amplitude of low-frequency fluctuations (fALFF) and functional connectivity (FC).

**Results:**

TTNS significantly enhanced clinical outcomes, reducing 24 h urinary frequency from 15.53 ± 2.97 to 12.87 ± 2.88 (*p* < 0.001) and decreasing OABSS from 9.67 ± 1.50 to 8.53 ± 1.41 (*p* < 0.001). Neuroimaging indicated improvements were supported by notable increases in neural activity during bladder fullness, particularly in the fALFF of the left cerebellum posterior lobe (*p*FWE < 0.001) and right medial frontal gyrus (*p*FWE *=* 0.004). Additionally, FC between the left cerebellum posterior lobe and left fusiform gyrus was strengthened (*p*FWE *=* 0.005). Correlation analysis revealed that higher functional connectivity was associated with lower OAB symptom scores (*p* = 0.001). Importantly, Baseline fALFF differences between bladder states no longer met the corrected significance threshold after treatment (*p* < 0.05).

**Conclusion:**

In this cohort, 1 month of TTNS was associated with reductions in urinary frequency and OABSS and with changes in fALFF and FC. These preliminary findings do not establish neuroplasticity, normalization, or a causal TTNS-specific mechanism and require confirmation in adequately powered sham-controlled studies.

**Clinical trial registration:**

https://www.chictr.org.cn/hvshowprojectEN.html?id=300046&v=1.0, Identifier: ChiCTR2600122960

## Introduction

Overactive bladder (OAB) is a prevalent chronic condition marked by urinary urgency and frequency, significantly diminishing patients’ quality of life (Shaw and Gibson, 2023; Filipetto et al., 2014). Globally, OAB’s prevalence remains high: a 2022 systematic review reported an overall prevalence of about 20% (Zhang et al., 2025). Recent epidemiological data from China reflect a similar pattern, with prevalence rising notably with age, thereby imposing a significant burden on the healthcare system (Zhang et al., 2025; Irwin et al., 2009). While the incidence of clinical symptoms like urgency and frequency is well-documented, the incidence and characteristics of underlying central nervous system dysfunction in OAB patients remain insufficiently explored. This gap in knowledge limits our understanding of the mechanisms and hinders the optimization of treatment strategies.

Transcutaneous Tibial Nerve Stimulation (TTNS) has emerged as an effective non-pharmacological treatment for OAB (Gaziev et al., 2013; Bressington et al., 2023). This therapy modulates the “nerve-bladder” reflex arc, where stimulating tibial nerve afferent fibers indirectly regulates the excitability of the sacral micturition center, thus suppressing detrusor overactivity (Al-Danakh et al., 2022). Evidence suggests that the therapeutic effects of electrical stimulation therapy may extend beyond local spinal modulation to include neuroplastic changes in brain activity and functional connectivity (Luo and Liao, 2025). Research on poor prognosis in other neurological conditions, such as post-stroke OAB, implies that interventions targeting central pathways might offer more sustained efficacy (Bapir et al., 2022). However, the specific central mechanisms of TTNS remain unclear.

Previous neuroimaging studies have limitedly elucidated the central mechanisms of TTNS. Functional magnetic resonance imaging (fMRI) research has confirmed that a single TTNS session activates multiple brain regions, such as the brainstem and sensorimotor cortex (Krhut et al., 2023). Additionally, functional near-infrared spectroscopy (fNIRS) studies have shown significantly increased prefrontal cortex activity during acute TTNS in treatment-responsive OAB patients, correlating with symptom improvement (Li et al., 2023). Regarding FC, fMRI studies have demonstrated enhanced coordination between multiple brain regions. Notably, the increased FC among areas such as the primary motor cortex, the periaqueductal gray, and the pontine micturition center may constitute a key mechanism underlying the improvement of bladder function by TTNS (Liu et al., 2025). Animal experiments further support the modulatory role of the tibial nerve pathway on brain regions involved in urination, such as the pontine micturition center (Lyon et al., 2015; Coolen et al., 2023).

Despite advancements, critical controversies and limitations remain in current research. Firstly, most studies have concentrated on healthy volunteers or the acute effects of a single stimulation session, making it difficult to generalize their findings to longer-term changes in brain function in patients with OAB (Ju et al., 2025; Girtner et al., 2021). Longitudinal studies are therefore needed to evaluate cumulative treatment-associated changes in this clinical population. Secondly, existing methodologies struggle to effectively differentiate between general somatosensory activation induced by TTNS and bladder-specific sensory modulation. For example, existing methods have difficulty distinguishing general somatosensory activation induced by TTNS from bladder-related sensory modulation; activity observed during stimulation may reflect limb sensory input rather than modulation of bladder-control networks (Smith, 2018). To address these gaps, it is essential to employ metrics that capture both regional and network-level neural activity. The fractional amplitude of low-frequency fluctuations (fALFF) indicates the intensity of regional spontaneous neural activity (Zhang et al., 2021), whereas functional connectivity (FC) measures the temporal correlation or functional synergy between different brain regions (de Lange et al., 2023). These complementary metrics were used to examine regional and network-level changes after 1 month of TTNS. The full-bladder versus empty-bladder comparison was intended to examine state-dependent patterns potentially related to treatment, but it cannot by itself isolate TTNS-specific effects in the absence of a control group.

## Methods

### Study population

This prospective longitudinal self-controlled study was conducted from March 17, 2024, to June 21, 2025, with follow-up concluding on July 25, 2025, at the China Rehabilitation Research Center. Fifteen adults with a neurological condition (spinal cord injury or multiple sclerosis) and symptom-defined OAB were enrolled. OAB was defined clinically by urinary urgency, usually accompanied by frequency and/or nocturia, with or without urgency urinary incontinence, after exclusion of urinary tract infection, urinary obstruction, and other relevant pathology. Inclusion criteria required participants to be aged 18–75 years, provide informed consent, maintain a 3-day voiding diary showing an average of more than 8 daytime voids and more than 2 nighttime voids per 24 h, agree to discontinue the use of medications and other relevant therapeutic measures affecting lower urinary tract symptoms 1 month prior to the trial, and communicate effectively with researchers. Exclusion criteria encompassed pregnancy or lactation, uncontrolled urinary tract infection, urinary obstruction, malignancy, psychiatric disorders, severe comorbidities, botulinum toxin treatment within the past 12 months, significant pelvic organ prolapse, participation in other clinical trials within 3 months, and any other conditions deemed unsuitable by the investigators. Data were collected prospectively through clinical interviews, voiding diaries, urodynamic studies, and fMRI to ensure consistency and completeness.

### Intervention protocol

The TTNS intervention was performed using a transcutaneous electrical nerve stimulation device (General Stim, Inc., Hangzhou, Zhejiang, China). The device was programmed at a frequency of 20 Hz, a pulse width of 200 μs and an adjustable current range of 0–30 mA, consistent with the previously published protocol for the same device (Li et al., 2023). To ensure reproducibility, the stimulation electrodes were placed specifically at the medial malleolus: The stimulator is positioned about two finger-widths below and behind the inner ankle bone, with the center of the lower patch aligned directly with the ankle bone. The treatment intensity was individualized for each participant by gradually increasing the current from 0 mA until the patient reported a strong but comfortable tingling sensation in the sole of the foot or the hallux, or until visible plantar flexion of the hallux occurred. The intensity was individually adjusted to be noticeable and comfortable for the patient, avoiding discomfort or strong muscle contractions. To exclude the impact of differences in treatment duration, the treatment cycle was strictly controlled for a total of 30 consecutive days, with each session lasting 1 h, once daily (totaling 30 h of stimulation over 1 month). For statistical analysis in this self-controlled design, outcome variables included changes in fALFF and FC from resting-state fMRI before and after treatment, along with cross-sectional differences in fALFF/FC between full and empty bladder states.

### MRI data acquisition and preprocessing

To maintain consistency, the scan of the full bladder was conducted first. Participants were instructed to drink 500–1,000 mL of water 1 h prior to the scan until they reported a strong desire to void (Level 4 on the Urinary Sensation Scale). Following the completion of the first session, participants were instructed to void completely. A pre-specified 45 min interval was then applied uniformly before the empty-bladder scan to reduce immediate carryover from the full-bladder scan and voiding and to allow bladder sensation and physiological state to stabilize. This was a standardized operational interval, not a validated physiological washout period. The fMRI data were collected using a 3 T scanner (Philips Ingenia, Best, Netherlands). During the resting-state scans, participants were instructed to keep their eyes closed, stay awake, and refrain from structured thinking. High-resolution T1-weighted anatomical images were captured before the functional scans. The anatomical scanning parameters were as follows: field of view (FOV) = 256 × 256 mm^2^, matrix size = 256 × 256, slice thickness = 1 mm, time repetition (TR) = 7,100 ms, time echo (TE) = 3.2 ms, flip angle (FA) = 7°, voxel size = 1 × 1 × 1 mm^3^. The resting-state fMRI scans were acquired using echo-planar imaging sequence with the following imaging parameters: slice thickness = 3.5 mm, TR = 2000 ms, TE = 30 ms, FA = 90°, slice gap = 0.85, FOV = 224 × 224 mm^2^, matrix size = 64 × 64, voxel size = 3.5 × 3.5 × 3.5 mm^3^.

Functional image preprocessing was conducted using the Resting-State fMRI Data Analysis Toolkit plus (RESTplus, http://www.restfmri.net) in conjunction with Statistical Parametric Mapping (SPM12, http://www.fil.ion.ucl.ac.uk/spm). The preprocessing pipeline comprised several steps: The pipeline was executed as follows: Prior to analysis, the initial 10 time points of each scan were discarded to achieve magnetic equilibrium. Subsequently, slice timing correction was performed to address temporal discrepancies in slice acquisition, followed by head motion correction, during which any volume with excessive displacement (>2 mm translation or 2° rotation in any direction) was excluded. The data were then spatially normalized to the Montreal Neurological Institute space via unified segmentation of the T1-weighted anatomical images and resampled to a 3 × 3 × 3 mm^3^ isotropic voxel size. Spatial smoothing was subsequently applied with a 6 mm full-width at half-maximum Gaussian kernel. To enhance data quality, linear detrending removed slow drifts in the signal, and nuisance regression was employed to eliminate the effects of head motion parameters and physiological noise. Finally, temporal filtering was also applied during the functional connectivity analysis.

fALFF and FC were calculated using the DPABI software package. For both whole-brain fALFF comparisons and seed-based ROI-to-voxel FC analyses, a voxel-level cluster-forming threshold of *p* < 0.001 (uncorrected) and a cluster-level family-wise error (FWE)-corrected threshold of *p* < 0.05 were applied. The covariates considered were age, sex, etiology, disease duration, AIS grades, OABSS, and prior treatment history. Correlation analysis was used to examine the association between treatment-related FC changes and OABSS changes.

### Ethic statement

The Ethics Committee of the China Rehabilitation Research Center approved the study protocol (approval number: CRRC-IEC-RF-SC-005-01). The trial adhered strictly to the principles of the Declaration of Helsinki. Before enrollment, all participants gave written informed consent, agreeing to the use of their anonymized data for research purposes. The informed consent process highlighted voluntariness, confidentiality, and the right to withdraw at any time, ensuring ethical compliance and protecting participants’ rights.

### Statistical analysis

Statistical analyses were conducted using SPSS version 26 (IBM, IL, United States). Normally distributed data are presented as mean ± standard deviation, and non-normally distributed data as median with interquartile range. Paired t-tests or non-parametric rank-sum tests were used as appropriate. Pearson or Spearman correlation analysis was used according to data distribution. Imaging multiple-comparison thresholds are specified above.

## Results

### Patient characteristics at baseline

Table 1 details the enrollment of fifteen patients with neurogenic OAB in this study. The group included 5 males (33.33%) and 10 females (66.67%), with an average age of 39.33 ± 13.20 years and an average disease duration of 2.67 ± 1.35 years. The neurological etiology was spinal cord injury in 13 participants (86.67%) and multiple sclerosis in two participants (13.33%). Among the 13 participants with spinal cord injury, three were classified as AIS grade B (23.08%), three as AIS grade C (23.08%), and seven as AIS grade D (53.85%). No participant had an AIS grade A injury; thus, all participants with SCI had incomplete spinal cord injuries. At baseline, patients showed severe OAB symptoms, with a mean OABSS of 9.67 ± 1.50. A 3 day voiding diary indicated frequent daytime voids (15.53 ± 2.97 episodes/24 h), urgency incontinence (1.53 ± 1.51 episodes/24 h) and nocturia (4.87 ± 1.46 episodes/24 h). Urodynamic studies revealed a significantly reduced maximum cystometric capacity (274.53 ± 36.75 mL), and 46.67% of the patients exhibited detrusor overactivity. Urodynamics was not repeated after treatment, and no other baseline urodynamic variables were available for the present analysis. Of the patients, 20.00% had previously undergone pelvic floor rehabilitation, while 80.00% had not received any relevant treatment.

**Table 1:** Baseline demographic and clinical characteristics of the patients.

Variables	Value (*n* = 15)
Gender	
Male (*n*, %)	5 (33.33)
Female (*n*, %)	10 (66.67)
Age (years)	
Mean age ± SD	39.33 ± 13.20
Pathogenesis	
Spinal cord injury (*n*, %)	13 (86.67)
AIS grade among participants with SCI (*n* = 13), *n* (%)	
A	0 (0)
B	3 (23.08)
C	3 (23.08)
D	7 (53.85)
E	0 (0)
Multiple sclerosis (*n*, %)	2 (13.33)
Others (*n*, %)	0 (0)
Disease duration (years)	
Mean duration ± SD	2.67 ± 1.35
Overactive Bladder Symptom Score	
Baseline score ± SD	9.67 ± 1.50
3-day voiding diary	
Voids/24 h	15.53 ± 2.97
Urgency incontinence episodes/24 h	1.53 ± 1.51
Nocturia episodes/24 h	4.87 ± 1.46
Urodynamic parameters	
Maximum cystometric capacity (mL)	274.53 ± 36.75
Detrusor overactivity (*n*, %)	7 (46.67)
Past treatment history	
Prior pelvic floor rehabilitation (*n*, %)	3 (20.00)
None (*n*, %)	12 (80.00)

### Clinical outcomes following transcutaneous tibial nerve stimulation

Following the complete treatment course, key clinical outcomes showed significant improvements, as detailed in Table 2. The primary outcome, 24 h urinary frequency, significantly decreased from 15.53 ± 2.97 to 12.87 ± 2.88 episodes (*p* < 0.001). Similarly, the total OABSS score significantly dropped from 9.67 ± 1.50 to 8.53 ± 1.41 (*p* < 0.001). Although daily urgency incontinence episodes decreased from 1.53 ± 1.51 to 1.20 ± 1.08, this reduction did not achieve statistical significance (*p* = 0.096).

**Table 2:** Comparison of clinical and functional outcomes before and after treatment.

Outcome Measures	Baseline	Post-treatment	*p*-value
Clinical outcomes			
Voids/24 h	15.53 ± 2.97	12.87 ± 2.88	<0.001
Urgency incontinence episodes/24 h	1.53 ± 1.51	1.20 ± 1.08	0.096
Overactive Bladder Symptom Score	9.67 ± 1.50	8.53 ± 1.41	<0.001
Functional magnetic resonance imaging			
Fractional amplitude of low-frequency fluctuations			
Left cerebellum posterior lobe	−0.54 ± 0.53	0.56 ± 0.44	<0.001
Right medial frontal gyrus	−0.52 ± 0.83	0.44 ± 0.75	0.004
Functional connectivity			
Left cerebellum posterior lobe-left fusiform gyrus	0.14 ± 0.24	0.39 ± 0.26	0.005

### Primary outcomes: brain functional changes in response to TTNS therapy

Exploratory pre-to-post fMRI analyses identified supra-threshold differences during the full-bladder condition, whereas no differences met the corrected threshold in the empty-bladder condition. Given the uncontrolled design, these differences are described as treatment-associated and cannot be attributed specifically to TTNS.

**Figure 1:**
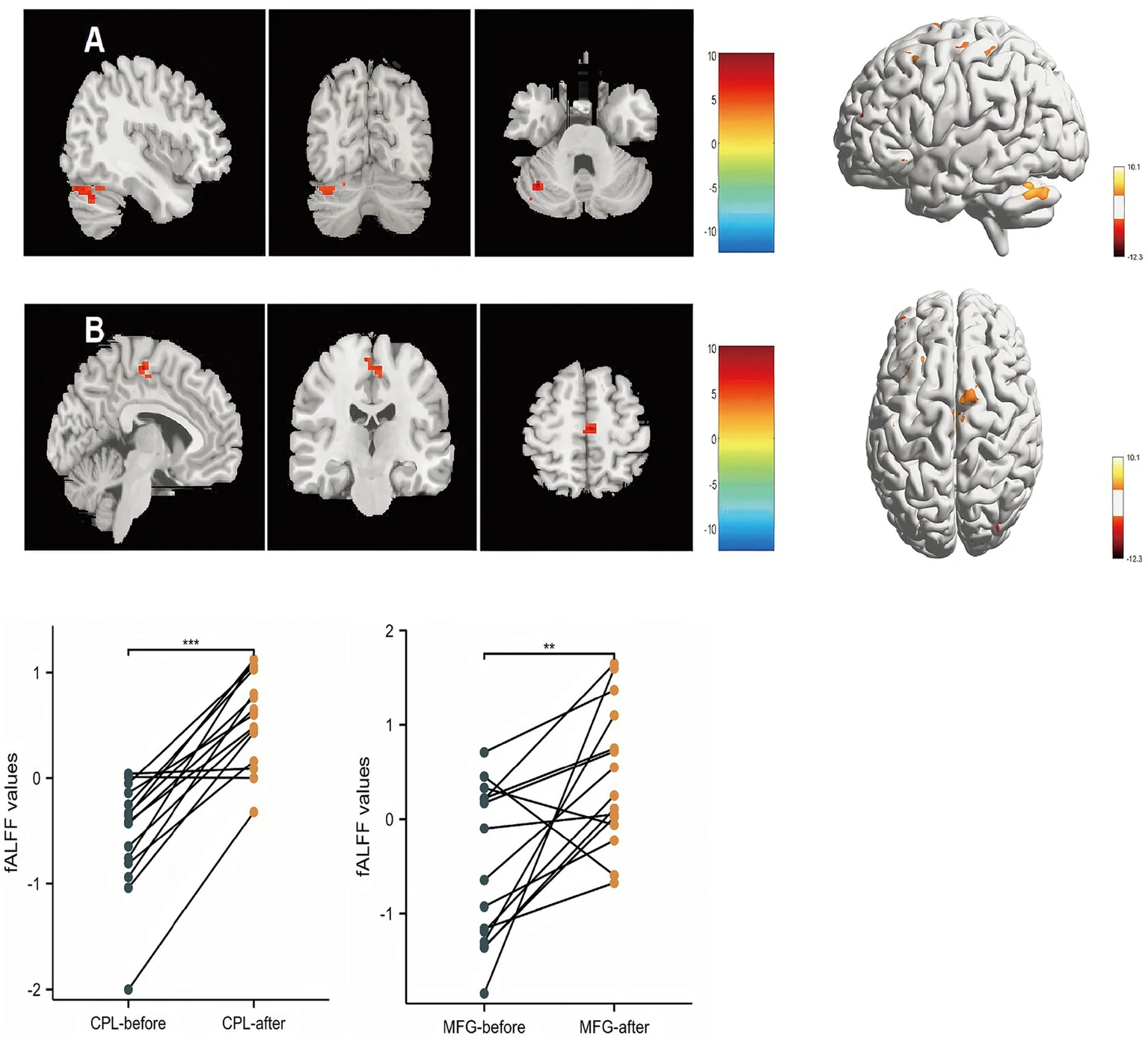
Significantly increased fALFF in left cerebellum posterior lobe **(A)** and right medial frontal gyrus **(B)** in OAB patients during the filling phase after TTNS treatment. The map of the brain regions with statistical differences between before and after TTNS treatment in OAB patients and three-dimensional distributions. CPL, cerebellum posterior lobe; MFG, medial frontal gyrus. **p < 0.01; **p < 0.001.

**Figure 2:**
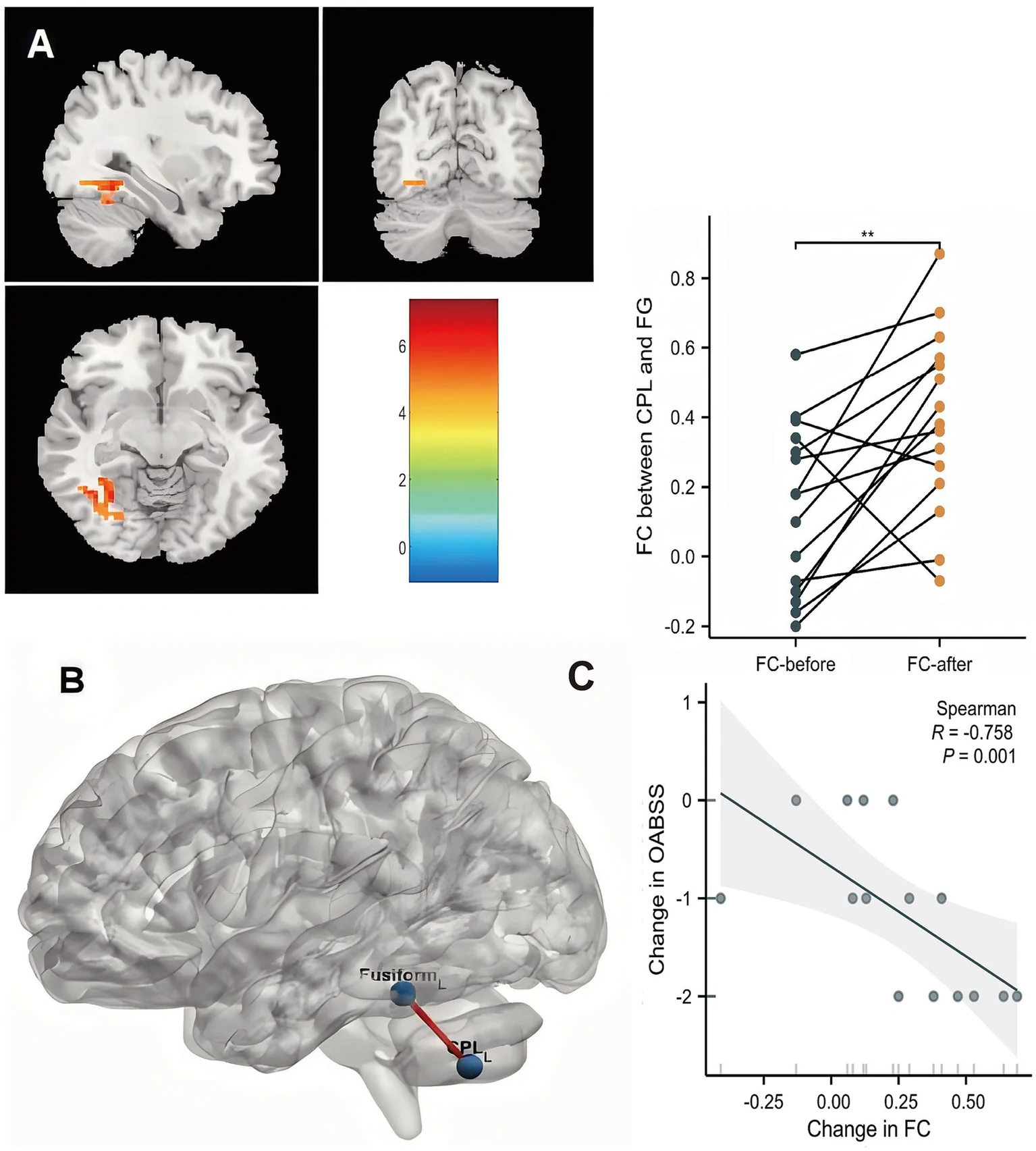
Significantly increased FC values between left cerebellum posterior lobe and left fusiform gyrus after TTNS treatment in OAB patients during the filling phase in ROI-voxel analysis (left cerebellum posterior lobe as a seed). **(A)** Red regions (left fusiform gyrus) represent higher FC values in OAB patients after TTNS treatment and **(B)** three- dimensional distributions. **(C)** The correlation between the change in FC values (post − pre) between the left cerebellum posterior lobe and left fusiform gyrus and the change in OABSS (post − pre). FC, functional connectivity; FG, fusiform gyrus; OABSS, Overactive Bladder Symptom Score; **p < 0.01.

During bladder fullness, we observed a notable increase in neural activity, indicated by a rise in the fALFF within the left cerebellum posterior lobe (ROI: −39, −63, −30) (*p*FWE < 0.001, Figure 1A) and the right medial frontal gyrus (ROI: 3, −18, 57) (*p*FWE = 0.004, Figure 1B). Simultaneously, post-treatment FC values between the left cerebellum posterior lobe and the left fusiform gyrus were significantly enhanced (*p*FWE *=* 0.005, Figure 2A). This connectivity is depicted in the three-dimensional rendering (Figure 2B). Scatter plot illustrating the correlation between the change in FC values between the left cerebellum posterior lobe and left fusiform gyrus and the change in OABSS (*p* = 0.001, Figure 2C). Higher functional connectivity was associated with lower OAB symptom scores. These preliminary observations raise the possibility that TTNS may be associated with alterations in regional neural activity and inter-regional communication within brain networks involved in bladder sensation and control.

### State-specific brain responses before and after treatment

A direct comparison between the states of an empty and a full bladder further was used to examine state-dependent patterns before and after treatment.

**Figure 3:**
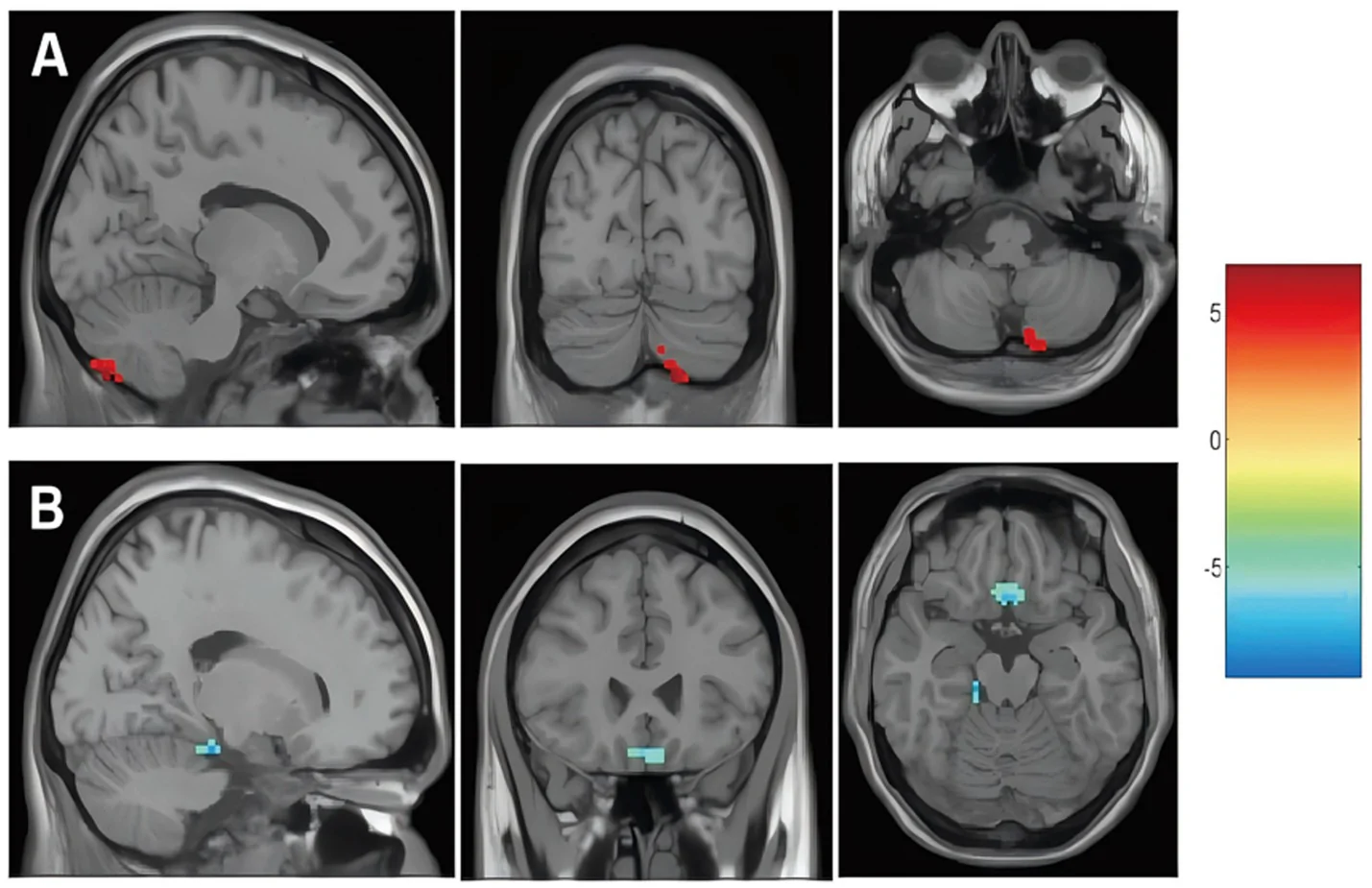
The map of the brain regions with statistical differences in fALFF changes in OAB patients when the empty bladder state compared to the full bladder state before TTNS treatment. Increased fALFF was found in **(A)** left cerebellum posterior lobe and **(B)** right medial frontal gyrus and Brodmann area 25 when the empty bladder state compared to the full bladder state before TTNS treatment.

At baseline, notable differences in fALFF were identified between the two states. Specifically, fALFF in the left cerebellum posterior lobe (ROI: −3, 21, −18) was higher during the state of emptiness (*p*FWE < 0.05, Figure 3A). In contrast, fALFF in the region encompassing the right medial frontal gyrus (ROI: 6, −75, −39) and Brodmann area 25, part of the ventromedial prefrontal cortex, was lower during fullness compared to emptiness (*p*FWE < 0.05, Figure 3B).

After TTNS, the previously observed fALFF differences between empty-bladder and full-bladder states in these regions no longer met the corrected significance threshold. Loss of statistical significance does not demonstrate normalization and may also reflect limited power or test–retest variability.

## Discussion

In this prospective longitudinal self-controlled trial, we assessed the impact of one-month TTNS on clinical symptoms and central brain function in patients with refractory neurogenic overactive bladder. The study’s rigorous within-subject design, enhanced by an innovative cross-state fMRI comparison between full and empty bladder conditions, ensured precise attribution of observed effects to the intervention. TTNS significantly improved clinical outcomes, reducing 24 h urinary frequency from 15.53 ± 2.97 to 12.87 ± 2.88 episodes and lowering the OABSS from 9.67 ± 1.50 to 8.53 ± 1.41. Additionally, TTNS was associated with altered central processing of bladder fullness, as evidenced by increased fALFF in the left cerebellar posterior lobe and right medial frontal gyrus, alongside strengthened functional connectivity between the left cerebellum and left fusiform gyrus. Notably, the pre-treatment differentiation in fALFF between empty and full bladder states was no longer observed after intervention. In the absence of a healthy control group, we cannot conclude that TTNS restores normative brain-bladder interactions; rather, these findings indicate an attenuation of the baseline bladder-state signal imbalance. While this pattern is compatible with improved central integration of visceral input, the analyses derive from a small exploratory cohort (*n* = 15). Therefore, these results should be interpreted as preliminary and hypothesis-generating, with inherent risks of both false-positive and false-negative findings.

Our study demonstrated significant clinical improvements, evidenced by reductions in 24 h urinary frequency and OABSS. This clinical-functional correlation is supported by Sethi and Peters (2025) and Shah et al. (2025). However, unlike Lucente et al. (2025), we did not observe a significant reduction in urinary incontinence frequency after 1 month of TTNS in OAB patients. This discrepancy may be due to the shorter treatment duration and the less severe incontinence in our patient population compared to theirs.

This pilot study provides preliminary evidence that 1 month of TTNS modulates activity and connectivity within key brain regions involved in bladder control, which appears to correlate with symptomatic improvement in patients with overactive bladder. The increase in neural activity observed in the medial frontal gyrus and the posterior lobe of the cerebellum after TTNS treatment is consistent with previous neuroimaging studies on TTNS’s central mechanisms. Evidence increasingly highlights the crucial role of frontal lobe dysfunction in OAB pathophysiology. For example, studies have noted significant reductions in the fALFF, regional homogeneity (ReHo), and degree centrality within the medial frontal gyrus of patients with refractory OAB (Duan et al., 2025). These findings suggest impaired function and connectivity in this region, underscoring its potential as a therapeutic target. Gao and Liao (2021) confirmed, through fMRI, abnormalities in ReHo and FC in the frontal lobes of OAB patients, indicating that frontal lobe dysfunction may contribute to voiding control disorders. The frontal lobe, a hub for higher-order cognitive and behavioral control, is crucial for the cognitive inhibition necessary for bladder control. Supporting this, Wang et al. found that higher functional connectivity was associated with lower OAB symptom scores, suggesting that diminished function in this area may impair bladder control and worsen symptoms (Biao et al., 2021). Our findings align with the fNIRS study by Li et al. (2023), which also reported increased prefrontal cortex activity during TTNS that correlated with symptomatic improvement. Our findings are compatible with involvement of frontal control systems, but the present design cannot determine whether the changes reflect a TTNS-specific mechanism.

The cerebellum’s role in bladder control is increasingly recognized. While traditionally linked to motor coordination, neuroimaging evidence shows that the cerebellum, through its extensive connections with the brainstem and higher cortical areas, significantly integrates bladder sensation and motor function and modulates voiding reflexes (Baumann and Mattingley, 2022; Tsuchiya et al., 2020). fMRI studies reveal dynamic changes in functional activity across cerebellar sub-regions as the bladder transitions from empty to full, highlighting the cerebellar network’s sensitivity to bladder filling and its involvement in the storage phase (Mazeaud et al., 2024). The prevailing view is that the cerebellum aids in maintaining urinary storage primarily by modulating pelvic floor muscle tone (Bastide and Herbaut, 2020). Experimental and clinical data consistently support the cerebellum’s regulatory role, with increased bold signals during the storage phase and strong functional connections to sensory and motor control networks, suggesting dual regulation of bladder sensation and pelvic floor motor control (Mazeaud et al., 2024; Kreydin et al., 2020). Additionally, research indicates that the cerebellum may influence brainstem and forebrain micturition centers through direct or indirect pathways, thereby modulating voiding reflexes (Bastide and Herbaut, 2020). Neuro-modulation studies further show that electrical stimulation therapies for OAB can activate multiple brain regions, including the cerebellum and prefrontal cortex, helping to restore sensory regulation and alleviate OAB symptoms (Babadi et al., 2021).

A novel finding of this study is the significantly enhanced FC between the posterior lobe of the cerebellum and the fusiform gyrus following TTNS treatment. In untreated OAB patients, the brain-bladder control network is dysregulated. The cerebellar posterior lobe, which acts as a sensorimotor coordinator, may fail to effectively process signals from higher cortical areas, resulting in insufficient inhibitory control over the detrusor muscle. The fusiform gyrus, although primarily involved in higher-order visual processing, also plays a role in sensory integration and emotional regulation (Keller et al., 2017). In OAB, aberrant bladder sensation signals can lead to abnormal activity in brain regions involved in sensory integration and emotional response, such as the fusiform gyrus. The TTNS-induced enhancement of connectivity between these regions may have several implications: First, it may indicate a strengthening of the sensorimotor coordination circuit, allowing the brain to interpret bladder sensations more accurately via the fusiform gyrus and achieve more effective integration of motor commands through the cerebellum. Second, it may reflect a normalization of the emotional stress response. By mitigating the threat posed by abnormal bladder sensations, TTNS reduces anxiety levels associated with symptoms, leading to a more stable emotional response to bladder sensation and improved cognitive regulation of voiding reflexes by the cerebellum. However, the increased FC between the posterior cerebellum and fusiform gyrus should be regarded as an exploratory finding. Although the cerebellum contributes to sensorimotor and visceral integration and the fusiform gyrus is classically involved in higher-order visual processing (Mazeaud et al., 2024; Bastide and Herbaut, 2020; Keller et al., 2017), neither region is a core node of the canonical supraspinal micturition circuit centered on the periaqueductal gray (PAG) and pontine micturition center (PMC). We did not observe corrected treatment-associated changes in the PAG or PMC. This absence may reflect the small sample, seed selection, or limited sensitivity and should not be interpreted as evidence that these regions were uninvolved. The physiological meaning of cerebellum–fusiform connectivity in bladder control therefore remains uncertain and requires replication.

Our findings differ from the acute brainstem and sensorimotor responses reported by Krhut et al. (2023), possibly because of differences in population, stimulation duration, and imaging paradigm. The full- versus empty-bladder comparison allowed examination of state dependence, but without sham stimulation or healthy controls it cannot distinguish TTNS-specific effects from general somatosensory input, expectancy, natural history, or test–retest variability. Consequently, proposals involving enhanced top-down frontal inhibition, cerebellar sensory processing, or downstream modulation of the PAG/PMC remain hypotheses rather than demonstrated mechanisms.

This study has several important limitations. First, the sample was small (*n* = 15), limiting statistical power and precision, reducing generalizability, and increasing the risks of both false-positive and false-negative findings; the results should therefore be regarded as preliminary. Second, the lack of sham/placebo, untreated, and healthy control groups prevents separation of TTNS-specific effects from placebo effects, natural history, repeated-testing effects, or regression to the mean and precludes claims of normalization. Third, although all participants with SCI had incomplete injuries (AIS grades B–D), the small numbers within each AIS subgroup precluded reliable adjustment for AIS grade in the imaging analyses. Neurological heterogeneity across AIS grades may nevertheless have influenced the observed brain activity and functional connectivity, and AIS classification does not directly establish the integrity of bladder-specific ascending or descending pathways. Fourth, only maximum cystometric capacity and detrusor over-activity were available from baseline urodynamics, and no post-treatment urodynamic assessment was performed; therefore, urodynamic treatment effects cannot be evaluated. Finally, the mean 1.14-point OABSS reduction was below the published three-point clinically important threshold (Gotoh et al., 2011). Larger, adequately powered, sham-controlled studies with detailed neurological phenotyping, comprehensive baseline and follow-up urodynamics, and longer follow-up are needed.

## Conclusion

In this small uncontrolled cohort, 1 month of TTNS was associated with reductions in urinary frequency and OABSS and with changes in fALFF and FC during bladder fullness. The findings are preliminary and do not establish neuroplasticity, normalization of brain–bladder interactions, or a TTNS-specific causal mechanism. Larger sham-controlled studies are required to determine reproducibility and clinical relevance.

## Data Availability

The raw data supporting the conclusions of this article will be made available by the authors, without undue reservation.
